# Association between central thyroid hormone dysregulation and chronic kidney disease in type 2 diabetes: a cross-sectional study

**DOI:** 10.1080/0886022X.2026.2731649

**Published:** 2026-09-18

**Authors:** Heming Huang, Yanjia Shi, Ping Fang, Sijing Tang, Wei Xu, Chenxia Zhou, Yun Zhou, Keqin Zhang, Ying Xue

**Affiliations:** Department of Endocrinology and Metabolism, Tongji Hospital, School of Medicine, Tongji University, Shanghai, China

**Keywords:** Type 2 diabetes mellitus, chronic kidney disease, thyroid stimulating hormone, Central thyroid hormone resistance

## Abstract

This cross-sectional study investigated the association between central thyroid hormone resistance and chronic kidney disease (CKD) in patients with type 2 diabetes mellitus (T2DM) who have normal peripheral thyroid hormones. The study included 2,759 T2DM patients with normal serum free triiodothyronine (FT3) and free thyroxine (FT4) levels. Central thyroid sensitivity was quantified using the Thyroid Feedback Quantile-based Index (TFQI), thyrotrophic thyroxine resistance index (TT4RI), TSH Index (TSHI), and FT3/FT4 ratio. Utilizing multivariable logistic regression and restricted cubic splines (RCS), we found that elevated TSH levels and increased central thyroid hormone resistance (indicated by higher TFQI, TT4RI, and TSHI) were independently associated with an increased risk of CKD. Notably, RCS analysis revealed a significant non-linear relationship (*p* < 0.001). Participants in the highest TSH quartile exhibited a 1.5-fold increased risk of CKD in multivariable regression (OR 1.50, 95% CI 1.08–2.07). Crucially, this association remained robust after propensity score-matched analyses (*n* = 684 pairs, OR 1.66, 95% CI 1.21–2.27). In conclusion, for T2DM patients with normal peripheral thyroid hormones, elevated TSH and reduced central thyroid hormone sensitivity are independently associated with a heightened risk of eGFR-defined CKD. These easily calculated indices may serve as valuable clinical biomarkers for early renal risk stratification.

## 1. Introduction

Chronic kidney disease (CKD) is one of the common and serious chronic complications of type 2 diabetes mellitus (T2DM), affecting a substantial proportion of patients and markedly increasing the risk of cardiovascular morbidity and mortality [1–3]. Even mild renal impairment has been associated with adverse cardiovascular outcomes in individuals with T2DM, highlighting the importance of early identification of high-risk patients [4–6]. Although traditional risk factors such as hyperglycemia, hypertension, and dyslipidemia contribute to CKD development [7], they do not fully explain the heterogeneity of renal outcomes observed in patients with T2DM [3], suggesting that unidentified pathophysiological mechanisms may contribute to the residual risk.

Thyroid hormones play an essential role in regulating energy metabolism [8], vascular function [9,10], and renal hemodynamics [11]. Previous studies have reported associations between overt thyroid dysfunction and impaired kidney function, including reduced glomerular filtration rate and increased risk of CKD [12,13]. However, evidence regarding the relationship between thyroid regulation and CKD in patients with normal peripheral thyroid hormone levels remains limited and inconsistent [14,15]. Most existing studies have focused on single measurements of thyroid-stimulating hormone (TSH) or peripheral thyroid hormones [16,17], often neglecting non-linear associations or the complex feedback regulation of the hypothalamic–pituitary–thyroid (HPT) axis. Moreover, alterations in thyroid function observed in patients with CKD have frequently been attributed to the low triiodothyronine (T3) syndrome associated with chronic illness, making it challenging to disentangle whether thyroid abnormalities represent a secondary consequence of renal dysfunction, or an independent pathogenic factor driven by dysregulation of central HPT control [18,19].

Recently, increasing attention has been paid to the concept of thyroid hormone sensitivity, which reflects the integrity of the central and peripheral feedback mechanisms of the HPT axis, rather than isolated hormone concentrations. Novel indices have been proposed, such as the thyroid feedback quantile-based index (TFQI), thyrotrophic thyroxine resistance index (TT4RI), free triiodothyronine to free thyroxine (FT3/FT4) ratio, and TSH index (TSHI), to characterize impaired thyroid hormone sensitivity [20]. These indices are considered more stable markers of the HPT axis set point and can capture regulatory abnormalities undetectable by standard hormone measurements [21]. However, the association between these indices and the risk of CKD in type 2 diabetic patients with normal peripheral thyroid function has not been systematically evaluated.

Therefore, the present study aimed to investigate the associations between serum TSH levels and thyroid hormone sensitivity indices and the risk of CKD in type 2 diabetic patients with normal peripheral thyroid hormone levels. We further explored potential non-linear relationships and conducted sensitivity analyses adjusting for circulating FT3 levels and propensity score matching to assess the robustness of the findings and to clarify the independent association of central thyroid regulation with the pathogenesis of T2DM-associated CKD, while acknowledging the potential complexity of the underlying mechanistic pathways.

## 2. Methods

### 2.1. Study design and population

This retrospective cross-sectional study was conducted at Tongji Hospital in Shanghai, China, between August 2016 and March 2025. We identified patients with T2DM who were diagnosed according to the American Diabetes Association criteria. The inclusion criteria were as follows [1]: patients aged ≥18 years [2]; confirmed diagnosis of T2DM [3]; normal peripheral blood thyroid hormone levels (FT3 and FT4); and complete clinical and laboratory records. Exclusion criteria included: a history of known thyroid disease, thyroid surgery, pituitary disorders, acute infection, malignancy, pregnancy, and current use of medications known to affect thyroid function (including thyroid hormone replacement or antithyroid drugs). Normal peripheral thyroid function was defined as FT3 and FT4 concentrations within the laboratory-specific reference ranges ([FT3: 3.5–6.5 pmol/L; FT4: 11.5–22.7 pmol/L]), regardless of serum TSH levels. The study protocol was approved by the Ethics Committee of Shanghai Tongji Hospital (K-W-2026-001), and the requirement for written informed consent was waived due to the retrospective nature of the study and the anonymized processing of data.

### 2.2. Data collection and clinical measurements

Demographic information, medical history, and medication use were extracted from electronic medical records. Data collected included age, sex, duration of diabetes, smoking status, history of hypertension and history of coronary heart disease (CHD). In this study, CHD was defined based on historical medical records rather than *de novo* screening. The diagnostic criteria included a documented history of myocardial infarction, prior coronary revascularization (percutaneous coronary intervention or coronary artery bypass grafting), coronary angiography demonstrating ≥ 50% stenosis in at least one major coronary artery, or a clinical diagnosis of angina supported by positive stress imaging, in accordance with established cardiovascular guidelines [22]. Blood pressure values were obtained from medical records. According to standard clinical practice, blood pressure was measured in the seated position after resting for at least 5 min. Fasting blood samples were collected in the morning after an overnight fast. Serum levels of FT3, FT4, and TSH were measured using chemiluminescent immunoassays. Additional laboratory measurements included glycated hemoglobin (HbA1c), lipid profiles, hemoglobin (HGB), albumin (ALB), serum uric acid (UA), C-reactive protein (CRP), and serum creatinine. All biochemical analyses were performed using standardized methods at Shanghai Tongji Hospital. All biochemical and clinical laboratory measurements were performed using standardized clinical analyzers from Beckman Coulter, Inc. (Brea, CA, USA). Specifically, biochemical parameters—including but not limited to HbA1c, lipid profiles, and serum creatinine—were measured using an AU5800 automatic biochemical analyzer; thyroid function parameters (TSH, FT3, and FT4) were determined using a UniCel Dxl 800 immunoassay system; and complete blood counts were analyzed using a DxH 900 automated hematology analyzer.

### 2.3. Assessment of renal function and definition of CKD

Renal function was assessed using the estimated glomerular filtration rate (eGFR), which was calculated based on serum creatinine using the Chronic Kidney Disease Epidemiology Collaboration (CKD-EPI) Equation [23]. CKD was defined as an eGFR < 60 mL/min/1.73 m^2^, in accordance with international clinical guidelines [24]. However, due to the retrospective nature of the electronic medical records, microalbuminuria data were not systematically available for all patients and were therefore not incorporated into the diagnostic criteria for this study.

### 2.4. Thyroid hormone sensitivity indices

To evaluate central and peripheral thyroid hormone sensitivity, TFQI, TT4RI, FT3/FT4 ratio, and TSHI were calculated as previously described [20,25–27], with the cumulative distribution functions for TFQI derived from the empirical distribution of our study cohort. TFQI reflects the relative position of individual FT4 and TSH levels within their respective population distributions, providing an integrated measure of pituitary feedback sensitivity. TT4RI and TSHI assess resistance to thyroid hormone at the pituitary level, whereas the FT3/FT4 ratio serves as an indicator of peripheral thyroid hormone conversion. The detailed formulas and physiological significance of these indices were provided in Table S1.

### 2.5. Statistical analysis

Normality of data distribution was assessed using the Shapiro-Wilk test and visual inspection of histograms. Continuous variables were expressed as median (interquartile range) due to skewed distributions, and categorical variables were presented as numbers of cases (percentages). Differences between groups were compared using the Mann–Whitney U test for continuous variables and the chi-square test for categorical variables. Multivariable logistic regression models were constructed to evaluate the associations between serum TSH levels, thyroid hormone sensitivity indices, and CKD. Covariates were selected *a priori* based on clinical relevance and included age, sex, duration of diabetes, smoking status, hypertension, CHD, systolic blood pressure (SBP) and low-density lipoprotein cholesterol (LDL-C). Restricted cubic spline (RCS) regression with five knots (placed at the 5th, 27.5th, 50th, 72.5th, and 95th percentiles) was applied to explore potential non-linear dose-response relationships between thyroid-related parameters and CKD risk. Given the U-shaped association observed in the RCS analysis, with a nadir at approximately 1.80 mIU/L, we designated the second quartile (Q2) as the reference group to avoid the artificial attenuation of effect estimates associated with extreme quartiles. To assess whether the association between TSH and CKD was independent of peripheral thyroid hormone levels, additional sensitivity models were fitted with further adjustment for serum FT3. Subgroup analyses were performed according to age, sex, and hypertension status. Interaction terms were tested to evaluate effect modification. To further minimize confounding factors, propensity score matching (PSM) was conducted to balance baseline characteristics between participants in the highest TSH quartile (Q4) and the reference quartile (Q2). Propensity scores were estimated using a logistic regression model incorporating the same covariates as in the multivariable models. A 1:1 nearest-neighbor matching algorithm without replacement was applied, with a caliper width equal to 0.2 of the standard deviation of the propensity score logit. Covariate balance was assessed using the standardized mean differences (SMD), with values < 0.1 indicating good balance. We designed this PSM as an extreme-case sensitivity analysis to rigorously isolate the risk of the highest TSH quartile (Q4) against the biological reference (Q2) under conditions of balanced covariates. While the full dose-response dynamic was primarily addressed through the aforementioned RCS analysis, this PSM approach serves to confirm the robustness of our main findings. All statistical analyses were performed using Python (version 3.x Python Software Foundation; https://www.python.org) and SPSS (version 27.0, IBM Corp.; https://www.ibm.com/products/spss-statistics). A two-sided P value < 0.05 was considered statistically significant.

## 3. Results

### 3.1. Baseline characteristics of the study population

A total of 2759 patients with T2DM and normal peripheral thyroid hormone levels were included in the analysis, among whom 375 (13.6%) were classified as having CKD. Baseline characteristics stratified by CKD status were summarized in Table 1. Compared with participants without CKD, those with CKD were significantly older (68.00 vs. 75.00 years, *p* < 0.001) and had a longer duration of diabetes, higher prevalence of hypertension and CHD, and higher SBP levels. Patients with CKD also exhibited significantly lower eGFR and markedly higher UA and CRP levels compared to non-CKD patients. Regarding thyroid parameters, patients with CKD presented significantly reduced serum FT3 levels (4.21 vs. 4.54 pmol/L, *p* < 0.001) and markedly decreased FT3/FT4 ratios compared to non-CKD patients. Serum TSH levels did not differ significantly between the CKD and non-CKD groups (1.91 vs. 1.78 mIU/L, *p* = 0.162). Regarding thyroid hormone sensitivity indices, the FT3/FT4 ratio was significantly lower in patients with CKD than in the non-CKD group (*p* < 0.001), indicating impaired peripheral tissue conversion of T4 to T3. Other indices reflecting central sensitivity (TFQI, TT4RI, and TSHI) did not show statistically significant differences between the groups in this unadjusted analysis (all *p* > 0.05). Additionally, Spearman correlation analysis was performed to evaluate the inter-relationships among raw thyroid hormones and the calculated sensitivity indices (Table S2).

**Table 1.** Baseline characteristics of patients with T2DM and normal peripheral thyroid hormone levels stratified by CKD status.

Variable	CKD (*n* = 375)	Non-CKD (*n* = 2384)	P-value
Age, years	75.00 (70.00–80.00)	68.00 (61.00–74.00)	<0.001
Sex			0.429
Male, n (%)	222 (59.2)	1466 (61.5)	
Female, n (%)	153 (40.8)	918 (38.5)	
HBP, n (%)	325 (86.7)	1738 (72.9)	<0.001
CHD, n (%)	260 (69.3)	1449 (60.8)	0.002
Smoking, n (%)	87 (23.2)	724 (30.4)	0.006
T2DM, years	10.00 (5.00–13.00)	8.00 (4.00–10.00)	<0.001
SBP, mmHg	139.00 (125.00–155.00)	136.00 (123.00–150.00)	0.019
DBP, mmHg	76.00 (69.00–84.00)	78.00 (70.00–85.00)	0.014
eGFR, mL/min/1.73 m^2^	48.50 (38.10–54.55)	90.50 (81.38–98.40)	<0.001
UA, μmol/L	430.00 (368.50–498.15)	331.00 (273.98–394.62)	<0.001
HbA1c, %	7.40 (6.70–8.30)	7.40 (6.70–8.50)	0.242
ALB, g/L	38.00 (35.60–40.90)	40.30 (37.90–42.70)	<0.001
HGB, g/L	123.00 (110.00–135.50)	134.00 (125.00–146.00)	<0.001
LDL-C, mmol/L	2.57 (2.00–3.26)	2.68 (2.06–3.29)	0.098
TG, mmol/L	1.50 (1.04–2.03)	1.38 (1.01–1.99)	0.090
CRP, mg/L	3.20 (1.00–8.70)	1.60 (0.60–4.50)	<0.001
FT3, pmol/L	4.21 (3.88–4.51)	4.54 (4.17–4.90)	<0.001
FT4, pmol/L	15.73 (14.25–17.52)	15.63 (14.31–17.20)	0.514
TSH, mIU/L	1.91 (1.17–2.99)	1.78 (1.21–2.64)	0.162
TFQI	0.06 (−0.21-0.27)	0.00 (−0.27-0.26)	0.081
TT4RI	29.62 (18.34–46.03)	28.10 (18.86–41.89)	0.093
FT3/FT4 ratio	0.27 (0.24–0.30)	0.29 (0.26–0.32)	<0.001
TSHI	2.79 (2.28–3.25)	2.71 (2.28–3.13)	0.059

Note: Continuous variables are expressed as median (interquartile range), and categorical variables are expressed as number (percentage).

Abbreviations: CKD, chronic kidney disease; CHD, coronary heart disease; T2DM, type 2 diabetes mellitus; HBP: High blood pressure; SBP, systolic blood pressure; DBP, diastolic blood pressure; eGFR, estimated glomerular filtration rate; UA, Uric Acid; HbA1c, glycated hemoglobin; ALB, albumin; HGB, hemoglobin; LDL-C, low-density lipoprotein cholesterol; TG, triglyceride; CRP, C-reactive protein; FT3, free triiodothyronine; FT4, free thyroxine; TSH, thyroid-stimulating hormone; TFQI, thyroid feedback quantile index; TT4RI, thyrotrophic thyroxine resistance index; TSHI, TSH index.

### 3.2. Association between serum TSH levels and CKD risk

**Figure 1.**
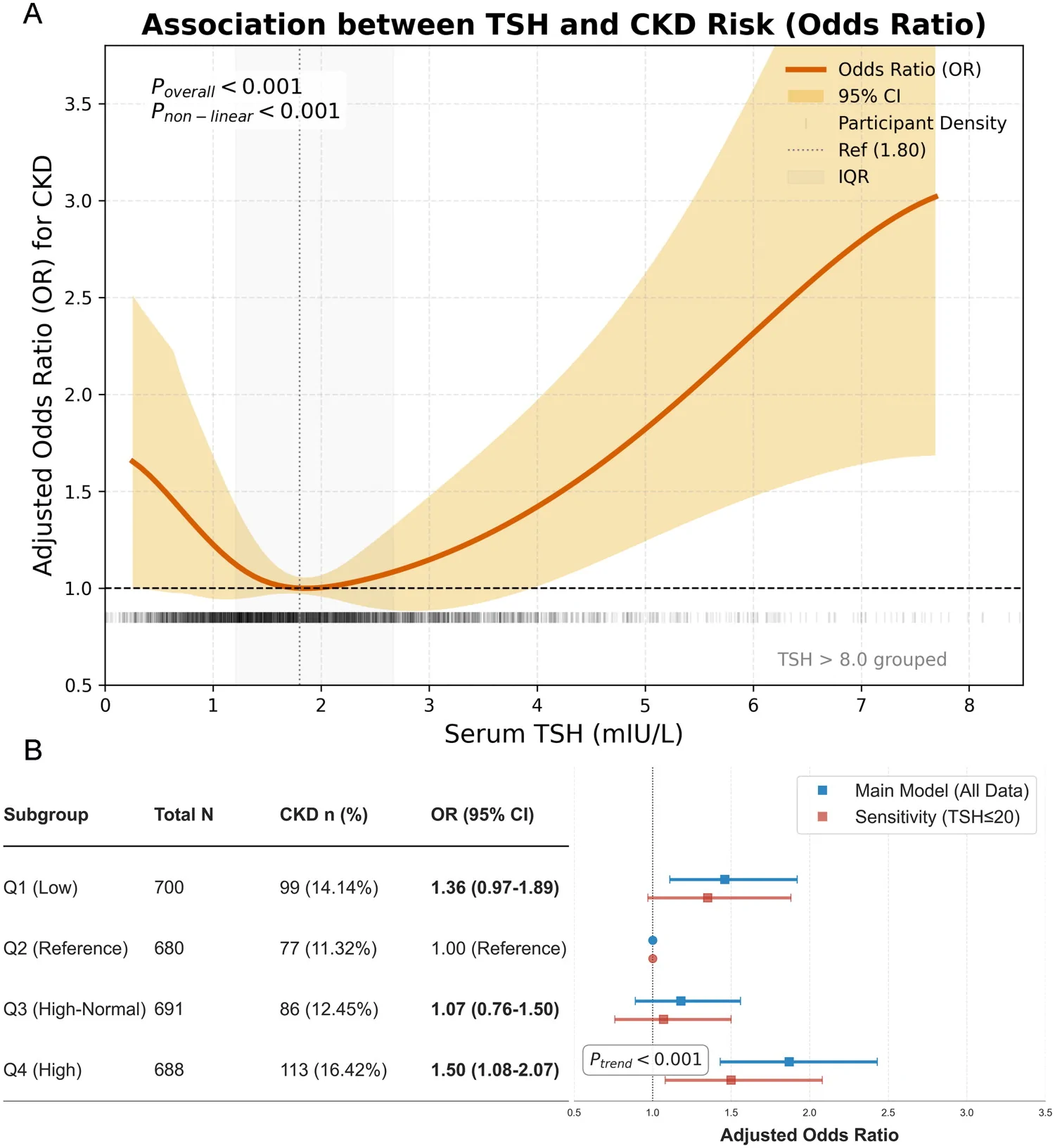
Association between serum TSH levels and the risk of CKD in patients with T2DM and normal peripheral thyroid hormone levels. (A) Restricted cubic spline (RCS) analysis showing the non-linear dose-response relationship between serum TSH and CKD risk. The solid red line and shaded area represent the multivariable-adjusted odds ratios (ORs) and 95% confidence intervals (CIs). The reference point (OR = 1.00) is set at the biological nadir of risk (1.80 mIU/L, dotted line). Rug plots along the x-axis illustrate the participant distribution. (B) Forest plot displaying the adjusted ORs and 95% CIs for CKD across TSH quartiles (Q1–Q4). The second quartile (Q2) served as the reference group. Blue squares represent the main model (all participants), while red squares represent the sensitivity analysis (excluding participants with TSH > 20 mIU/L). Models were adjusted for age, sex, history of hypertension, smoking status, history of CHD, diabetes duration, SBP, and LDL-C.

To comprehensively evaluate the relationship between TSH and CKD, we performed both continuous and categorical analyses. RCS regression was applied to explore the dose-response relationship between serum TSH and CKD risk. As shown in Figure 1A, a significant non-linear association was observed after multivariable adjustment (P for overall < 0.001; P for non-linearity < 0.001). The spline curve demonstrated that the lowest risk for CKD resides within the mid-normal range, with a progressive increase in risk as TSH levels approach the clinical thresholds.

To further evaluate these patterns, serum TSH levels were categorized into quartiles. As shown in Figure 1B, multivariable logistic regression analysis showed that, compared with the reference quartile (Q2), participants in the highest TSH quartile (Q4) had a significantly higher odds ratio for CKD (OR 1.50, 95% CI 1.08–2.07). Participants in the lowest quartile (Q1) exhibited a numerically higher risk (OR 1.36, 95% CI 0.97–1.89; *p* = 0.075). Sensitivity analyses excluding participants with TSH levels > 20 mIU/L yielded consistent results (Figure 1B), indicating that the observed associations were not driven by extreme values. Furthermore, sensitivity analysis using conventional clinical TSH reference ranges showed a consistent direction of risk with our primary analysis (Table S3), although the associations did not reach statistical significance (*p* > 0.05), likely due to the limited sample size and statistical power within the extreme TSH categories.

### 3.3. Thyroid hormone sensitivity indices and CKD risk

**Figure 2.**
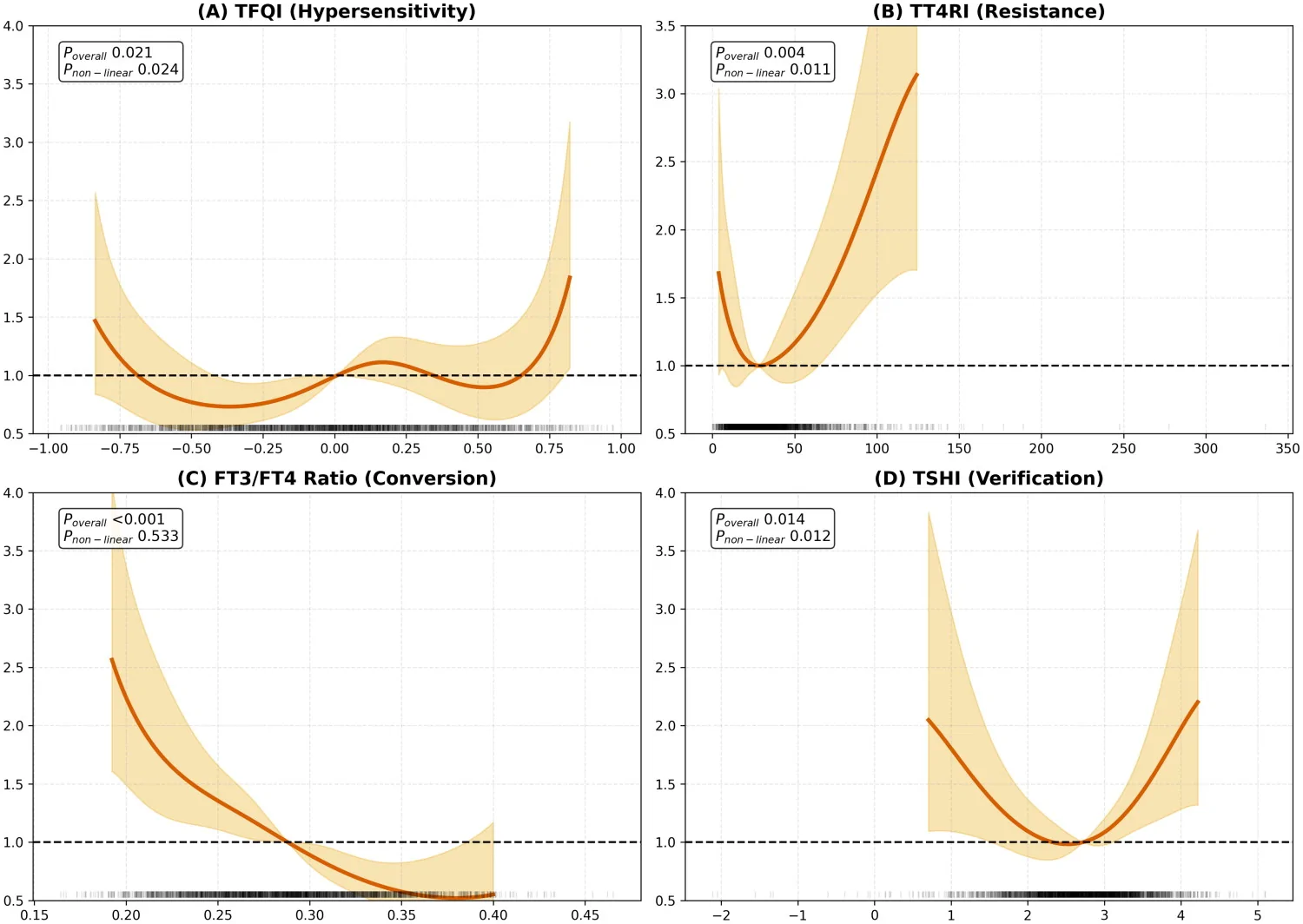
Dose-response associations of thyroid hormone sensitivity indices the risk of CKD in patients with T2DM and normal peripheral thyroid hormone levels. RCS regression models were used to analyze the associations of (A) TFQI, (B) TT4RI, (C) FT3/FT4 ratio, and (D) TSHI with the risk of CKD. The solid red lines represent multivariable-adjusted ORs, and light red shaded areas indicate 95% CIs. Rug plots along the x-axis illustrate the participant distribution. Dotted vertical lines indicate the reference values, while horizontal dashed lines represent OR =1.00. Models were adjusted for age, sex, hypertension history, smoking status, CHD history, duration of diabetes, SBP, and LDL-C.

RCS analyses revealed significant dose–response relationships between all four thyroid hormone sensitivity indices and CKD risk (Figure 2). Notably, TFQI, TT4RI, and TSHI exhibited nonlinear associations with CKD risk (all P for non-linear < 0.05), characterized by a pronounced increase in risk at the higher ranges of these indices. Conversely, the FT3/FT4 ratio exhibited a linear inverse correlation with CKD risk (P for non-linear = 0.533), with a monotonic increase in risk as the FT3/FT4 ratio declined.

### 3.4. Association of TSH with CKD independently of peripheral thyroid hormones

To assess whether the TSH-CKD association was independent of peripheral thyroid hormones, we performed a sensitivity analysis by additionally adjusting for serum FT3 levels. The association between elevated TSH levels (Q4 vs. Q2) and CKD risk remained statistically significant after adjusting for circulating FT3 levels (OR 1.53, 95% CI 1.10–2.13; *p* = 0.012). This indicates that the observed association between TSH and CKD is independent of peripheral thyroid hormone levels.

**Figure 3.**
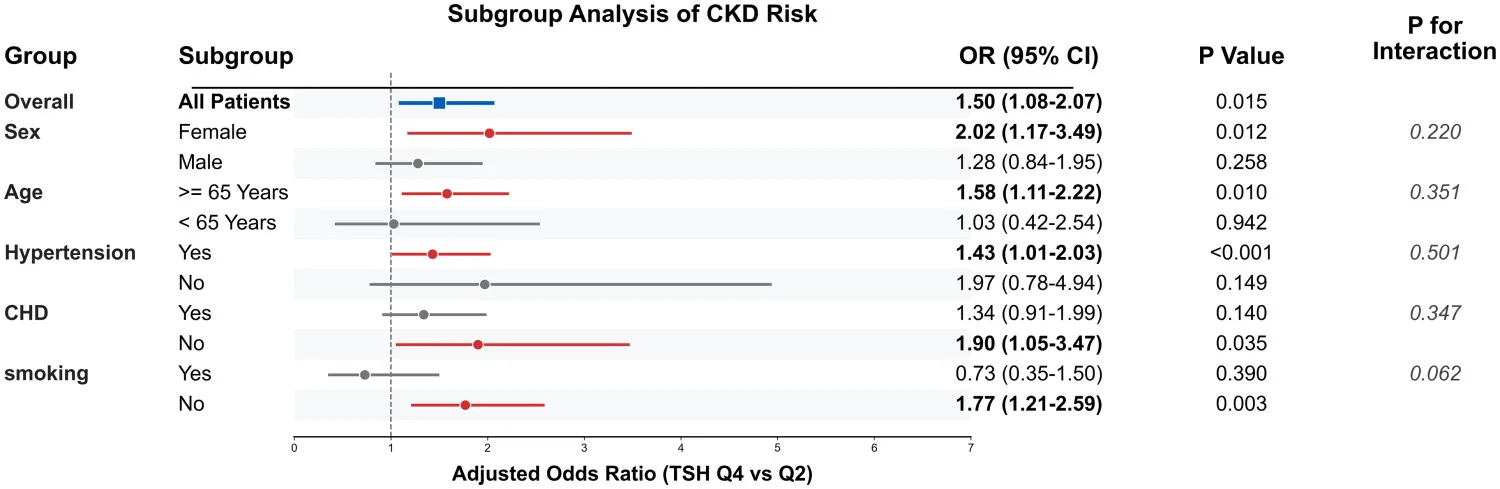
Subgroup analysis of the association between high TSH levels (Q4) and the risk of CKD compared with the reference group (Q2). Forest plots display the multivariable-adjusted ORs and 95% CIs for CKD in participants in the high TSH level group (Q4) versus the reference TSH level group (Q2), stratified by sex, age, smoking, CHD history, and hypertension history. Red markers indicate statistically significant associations (95% CI did not cross 1), whereas grey markers indicate non-significant results. P-values were used to test for interactions in heterogeneity between subgroups. ORs were derived from multivariable logistic regression models adjusted for age, sex, history of hypertension, smoking, diabetes duration, SBP, LDL-C, history of CHD. (Note: Stratification variables including age, sex, CHD history, smoking, or hypertension history were excluded from the models in their respective subgroup analyses).

Furthermore, we conducted subgroup analyses to explore potential heterogeneity in this association across clinically relevant strata (Figure 3). While the point estimates showed some variation, the positive association between higher TSH and CKD risk was generally consistent across subgroups defined by age, sex, smoking, and history of hypertension or CHD. Notably, interaction tests yielded no statistically significant heterogeneity (all P for interaction > 0.05). However, given that these multiple subgroup comparisons were exploratory and not corrected for multiple testing, these findings should be interpreted with caution as potential trends rather than definitive evidence of effect modification.

### 3.5. Propensity score matching (PSM) analysis

To further minimize confounding bias, PSM was employed. After 1:1 matching, 684 pairs of participants were included. All baseline covariates achieved excellent balance between the high-TSH group (Q4) and the reference groups (Q2), with all standardized mean differences < 0.1 (Figure S1). The propensity score distributions between the two groups showed sufficient overlap, confirming the adequacy of the common support region (Supplementary Figure 2). In this matched cohort, consistent with our primary analysis participants in the highest TSH quartile exhibited a significantly higher risk of CKD compared with the reference group (OR 1.66, 95% CI 1.21–2.27; *p* = 0.002) (Table 2). Furthermore, analysis based on clinically relevant TSH cutoffs within this same PSM-matched cohort provided additional confirmation of these findings (Table S4). These results futher corroborate the robustness of the observed association between elevated TSH levels and increased CKD risk, independent of established metabolic and clinical confounders.

**Table 2.** Baseline characteristics and CKD risk association analysis between the high TSH group (Q4) and reference group (Q2) in the propensity score-matched cohort.

Variables	Q2 (*n* = 684)	Q4 (*n* = 684)	SMD	P Value
Age (years)	70.0 (64.0–75.0)	70.0 (63.0–75.0)	0.023	0.665
Sex (male), n (%)	336 (49.1%)	336 (49.1%)	<0.001	1.000
HBP, n (%)	532 (77.8%)	523 (76.5%)	0.031	0.607
CHD, n (%)	398 (58.2%)	391 (57.2%)	0.021	0.743
T2DM, years	8.0 (5.0–10.0)	8.0 (4.0–10.0)	0.024	0.661
SBP (mmHg)	138.0 (125.0–153.0)	136.0 (125.0–154.0)	0.026	0.636
LDL-C (mmol/L)	2.7 (2.0–3.5)	2.7 (2.1–3.3)	0.004	0.945
FT3	4.5 (4.2–4.9)	4.5 (4.1–4.9)	0.032	0.558
CKD cases, n (%)	73 (10.7%)	113 (16.5%)	–	0.002
Logistic Regression (Post-Match)	OR (95% CI)			P Value
Q4 vs Q2	1.66 (1.21–2.27)			0.002

Note: Continuous variables are expressed as median (interquartile range), and categorical variables as number (percentage). Propensity score matching was performed at a 1:1 ratio.

Abbreviations: TSH, thyroid stimulating hormone; CKD, chronic kidney disease; SMD, standardized mean difference; OR, odds ratio; CI, confidence interval; CHD, coronary heart disease; T2DM, type 2 diabetes mellitus; SBP, systolic blood pressure; HBP: High blood pressure; LDL-C, low-density lipoprotein cholesterol.

### Statistical analysis

Continuous variables were compared between groups using the Mann-Whitney U test, while categorical variables were compared using the Chi-square test. A SMD < 0.1 indicated negligible intergroup differences. Logistic regression models were used to calculate the OR and 95% CI for the risk of CKD in the matched cohort.

## 4. Discussion

In this large cross-sectional study of patients with T2DM and normal peripheral thyroid hormone levels, we confirmed a significant nonlinear association between elevated serum TSH levels and increased risk of CKD. Importantly, multiple indices reflecting impaired thyroid hormone sensitivity, including TFQI, TT4RI, FT3/FT4 ratio, and TSHI, showed consistent and significant relationships with CKD risk. Notably, these associations remained robust after multivariable adjustment for cardiometabolic risk factors, additional adjustment for circulating FT3 levels, and propensity score matching. Notably, HbA1c levels were similar between the groups. However, this should be interpreted with caution. In patients with CKD, HbA1c often fails to accurately reflect true blood glucose levels. This is because conditions like anemia and shortened red blood cell lifespan—common in kidney disease—can cause HbA1c readings to appear falsely low, potentially masking differences in actual glycemic control [28,29]. These findings provide supportive evidence that central thyroid dysregulation may contribute to renal impairment in T2DM, independent of peripheral thyroid status. While single measurements of TSH capture isolated circulating concentrations, these integrated indices provide valuable incremental information by quantitatively characterizing the deviation of the HPT axis set point and relative central resistance, which cannot be captured by raw hormone levels alone.

Previous studies have frequently attributed thyroid abnormalities in CKD to the low T3 syndrome induced by chronic illness and catabolic states [19,30]. However, our results challenge this conventional perspective. Sensitivity analyses further adjusting for serum FT3 revealed only a slight attenuation of the TSH-CKD association, indicating that reduced peripheral thyroid hormone levels alone cannot fully explain the observed link between TSH and CKD risk. Instead, our findings support the concept that TSH itself may exert a direct effect on renal function in T2DM patients, or that dysregulation of the hypothalamic–pituitary–thyroid axis set-point plays an independent pathogenic role in CKD among diabetic individuals. The consistency of multiple thyroid hormone sensitivity indicators we observed with CKD risk in T2DM patients further strengthens this interpretation.

The precise mechanisms linking impaired central thyroid sensitivity (e.g. high-normal TSH and elevated TFQI) to the development of CKD in T2DM remain to be fully elucidated, but several interacting pathways are plausible. First, elevated TSH and subclinical hypothyroidism can induce systemic hemodynamic alterations, including increased peripheral vascular resistance and reduced renal perfusion, which may synergistically exacerbate diabetic ischemic microangiopathy [31]. Second, central HPT axis insensitivity might parallel peripheral tissue-specific resistance. Even with normal circulating FT3 concentrations, impaired local thyroid hormone receptor signaling or defective intracellular T3 utilization within renal cells could weaken tubular regenerative capacity and promote fibrosis [20]. Third, thyroid dysregulation and T2DM independently contribute to dyslipidemia and endothelial dysfunction, potentially forming a vicious cycle of metabolic injury [32]. Furthermore, unlike the hyperdynamic state in hyperthyroidism, which often induces temporary glomerular hyperfiltration, subclinical hypothyroidism directly drives a progressive decline in GFR [33]. Notably, the biological plausibility of these associations is supported by the identification of functional TSH receptors (TSHR) in human renal tissues, including glomerular mesangial cells and podocytes [34,35]. Mechanistically, functional TSH receptors (TSHR) are expressed in human renal tissues, including the glomeruli and tubular cells [34,35], providing a histological basis for the hypothesis that TSH may directly modulate renal function. While clinical observations have long suggested an interplay between thyroid and kidney disorders [18,27], the direction of this relationship has been clarified by recent genetic evidence. Specifically, Tsao et al. [16]. employed a multivariable Mendelian randomization approach to support a causal link, which aligns with our observed associations. Our findings further extend this by demonstrating non-linear dynamics of thyroid hormone resistance, consistent with the complex feedback patterns described in large population studies [25]. Furthermore, evidence further suggests that TSH can directly impair endothelial function through the induction of inflammatory cytokines such as TNF-α, which serves as a key mediator of renal vascular injury [36–39]. Nevertheless, these mechanisms remain partly hypothetical in our epidemiological context, and future experimental studies are warranted to validate these specific pathways.

Beyond the direct effects of TSH, our RCS analysis of TFQI offers a deeper physiological insight. Interestingly, the TFQI curve exhibited a U-shaped pattern. Positive TFQI values (indicating resistance) were strongly associated with CKD risk, while extremely negative TFQI values also showed an increasing trend in CKD risk. This finding supports the ‘hormonal homeostasis’ hypothesis, suggesting that any significant deviation from the HPT axis set-point toward either resistance or hypersensitivity [40,41] may be deleterious to renal health. Additionally, impaired central thyroid sensitivity (e.g. elevated TFQI) reflects a state of “thyroid hormone resistance,” which frequently coexists with insulin resistance and metabolic syndrome [20], thereby amplifying renal risk through synergistic metabolic dysregulation [42,43]. Although this study cannot directly validate these mechanisms, they provide a biologically plausible framework for the observed associations.

To ensure the robustness of our statistical analyses, we evaluated raw hormone concentrations (TSH, FT4) and calculated sensitivity indices (e.g. TFQI) in separate, parallel multivariable regression models. This approach effectively avoids potential internal variance inflation or mathematical coupling, ensuring that the independent associations observed for each metric are distinct. As detailed in our Spearman correlation matrix (Table S2), while these indices are related to raw hormone levels, they capture specific dimensions of HPT axis set-point deviations, providing incremental value for risk stratification beyond standard assays.

It is also noteworthy that while subgroup analyses across clinically relevant strata (e.g. sex, smoking) revealed some variation in point estimates, these findings were exploratory and did not reach statistical significance in interaction tests. Notably, we observed that the association between thyroid hormone resistance and CKD risk appeared more pronounced in females (OR: 2.02) compared to males (OR: 1.28), potentially suggesting sex-specific differences in thyroid hormone signaling or the differential impact of estrogen on renal hemodynamics. Conversely, the paradoxical association observed in the smoking subgroup (OR: 0.73 vs. 1.77 in non-smokers) warrants cautious interpretation, as it likely reflects the smaller sample size of this subgroup or potential residual confounding rather than a definitive reversal of risk. Given the potential for false-positive associations in multiple comparisons, these observations should be viewed as hypothesis-generating rather than definitive evidence of effect modification and thus warrant careful interpretation.

From a clinical perspective, our findings offer meaningful insights while warranting careful interpretation. Our restricted cubic spline analysis suggests that the lowest renal risk occurs within the mid-normal TSH range, with risk increasing at the extremes. To test this clinical utility, we performed a sensitivity analysis using conventional TSH reference ranges (<3.5, 3.5–5.0, and >5.0 mIU/L). Although these results (presented in Tables S3 and S4) showed a consistent direction of risk indicating that renal risk tends to become more evident at TSH levels exceeding the conventional upper limit (>5.0 mIU/L), the associations did not reach statistical significance (*p* > 0.05), likely attributable to the limited sample size in the extreme categories. Therefore, rather than serving as a rigid diagnostic threshold, these observations are hypothesis-generating and indicate that T2DM patients with TSH levels approaching or exceeding the upper conventional limits may warrant closer clinical attention and enhanced renal function monitoring. However, prospective studies are needed to determine whether such risk stratification improves long-term renal outcomes.

Several limitations should be acknowledged. First, the cross-sectional design precludes causal inference, making reverse causality a plausible alternative. The accumulation of systemic inflammatory mediators and uremic toxins, alongside altered protein binding of thyroid hormones in patients with established renal impairment, can directly disrupt the hypothalamic-pituitary-thyroid (HPT) axis. Consequently, these CKD-related physiological changes could potentially lead to the observed shifts in TSH and thyroid hormone sensitivity indices, rather than vice versa. This requirement for future prospective validation to definitively establish the directionality of the observed associations remains paramount [18,26,44]. Second, defining CKD solely by eGFR < 60 mL/min/1.73 m^2^ without albuminuria data is a major limitation, this approach inevitably misses early-stage CKD (stages G1–G2), introducing non-differential misclassification that biases our results toward the null, thus representing a conservative underestimation. Furthermore, reliance on a single creatinine measurement without 3-month confirmation fails to establish chronicity, meaning our conclusions apply strictly to eGFR-defined renal impairment. Third, residual confounding from unmeasured variables remains a concern, as we could not adjust for body mass index (BMI) or granular pharmacological data (e.g. dosage titrations of RAAS/SGLT2 inhibitors, or GLP-1 receptor agonists). Regarding markers like UA and ALB, we excluded them from our final models to avoid overadjustment bias, as they represent downstream physiological consequences of renal injury rather than upstream confounders. Finally, although PSM was employed, residual confounding cannot be fully ruled out, and thyroid function was assessed at a single time point. However, calculated indices of thyroid hormone sensitivity (e.g. TFQI) are generally considered more stable reflections of HPT axis set-point, potentially mitigating variability from single-point assessments. Given that TFQI values are derived from the study population’s empirical distribution, they currently serve as a robust research tool for identifying thyroid hormone resistance rather than a standardized diagnostic biomarker. Furthermore, our study spanned nearly nine years, a period characterized by significant evolution in T2DM management. Although the introduction of renoprotective agents (e.g. SGLT2 inhibitors, GLP-1 RAs) introduces potential temporal confounding, the persistent association observed throughout our study period suggests that the thyroid-metabolic-renal axis remains a clinically significant factor. Nonetheless, future studies should explicitly account for these evolving treatment patterns. Additionally, it is important to acknowledge that the TFQI in our study was derived from the empirical cumulative distribution function of our specific T2DM cohort. As a quantile-based metric, its absolute values are inherently cohort-dependent. Consequently, these values should not be directly compared across populations with distinct clinical characteristics, and future research utilizing standardized reference populations is required to establish more universally applicable diagnostic thresholds. Lastly, our study is limited by its single-center, ethnically homogeneous design, which may constrain the generalizability of our findings. Moreover, the high prevalence of comorbidities (CHD, hypertension) reflects a high-risk population with advanced metabolic disease, potentially limiting the representativeness for patients with early-stage T2DM. Therefore, our findings should be interpreted primarily within the context of high-risk T2DM patients in tertiary care settings, and future multi-center, multi-ethnic research is warranted to validate these results across broader populations.

In conclusion, this study demonstrates that elevated TSH levels and reduced thyroid hormone sensitivity remains independently associated with an increased risk of eGFR-defined CKD in T2DM patients, even when peripheral thyroid hormone levels are normal. These findings highlight the potential role of central thyroid dysregulation in the pathophysiology of CKD in diabetic patients and suggest that assessing thyroid regulatory function may provide additional insights for risk stratification in this high-risk population. Future longitudinal studies are warranted to clarify the temporal relationships and underlying mechanisms linking thyroid regulation to renal outcomes.

## Supplementary Material

Supplementary.docx

## Data Availability

The datasets generated and analyzed during the current study are available from the corresponding author upon reasonable request.

## List of abbreviations

CKD: Chronic kidney disease
T2DM: type 2 diabetes mellitus
TSH: thyroid-stimulating hormone
FT3: free triiodothyronine
FT4: free thyroxine
TFQI: thyroid feedback quantile-based index
TT4RI: thyrotrophic thyroxine resistance index
TSHI: TSH index
PSM: propensity score matching
OR: odds ratio
CIs: confidence intervals
HPT: hypothalamic–pituitary–thyroid
T3: triiodothyronine
CHD: coronary heart disease
HbA1c: glycated hemoglobin
HGB: hemoglobin
ALB: albumin
UA: uric acid
CRP: C-reactive protein
eGFR: estimated glomerular filtration rate
CKD-EPI: Chronic Kidney Disease Epidemiology Collaboration
SBP: systolic blood pressure
LDL-C: low-density lipoprotein cholesterol
RCS: restricted cubic spline
SMD: standardized mean differences
TSHR: TSH receptors.
