# Supplementary material for: Association between central thyroid hormone dysregulation and chronic kidney disease in type 2 diabetes: a cross-sectional study
Source: Ren Fail. 2026 Sep 18;48(1):2731649. doi: 10.1080/0886022X.2026.2731649 (PMC13591943; doi:10.1080/0886022X.2026.2731649)
Supplement: Supplementary file 1 [file IRNF_A_2731649_SM9377.docx]

Table S1. Formulas and definitions of thyroid hormone sensitivity indices.

| **Index** | **Abbreviation** | **Formula** | **Physiological Significance** |
| --- | --- | --- | --- |
| Thyroid Feedback Quantile-based Index | TFQI | CDF_TSH_ - (1 - CDF_FT4_) | Integrated measure of central thyroid hormone sensitivity; deviation from the population set point. |
| thyrotrophic thyroxine resistance index | TT4RI | FT4 ×TSH | Resistance to thyroid hormone at the pituitary level. |
| TSH Index | TSHI | Ln (TSH) + 0.1345×FT4 | Quantifies pituitary sensitivity to T4 inhibition (standardized for FT4 in pmol/L). |
| FT3-to-FT4 Ratio | FT3/FT4 | FT3 divided by FT4 | Peripheral deiodinase activity and T4-to-T3 conversion efficiency. |

Note: CDF: Cumulative distribution function (derived from the empirical distribution of the study population). TSH is measured in mIU/L; FT4 and FT3 are measured in pmol/L. ln: Natural logarithm. The coefficient 0.1345 in the TSHI formula is derived specifically for FT4 values in pmol/L.

Table S2. Spearman correlation matrix of raw thyroid hormones and calculated sensitivity indices.

| Variables | TSH | FT3 | FT4 | TFQI | TT4RI | TSHI | FT3/FT4ratio |
| --- | --- | --- | --- | --- | --- | --- | --- |
| TSH | 1.00 | 0.06 | -0.13 | 0.65 | 0.97 | 0.89 | 0.14 |
| FT3 | 0.06 | 1.00 | 0.19 | 0.18 | 0.10 | 0.14 | 0.55 |
| FT4 | -0.13 | 0.19 | 1.00 | 0.65 | 0.08 | 0.30 | -0.68 |
| TFQI | 0.65 | 0.18 | 0.65 | 1.00 | 0.80 | 0.91 | -0.40 |
| TT4RI | 0.97 | 0.10 | 0.08 | 0.80 | 1.00 | 0.97 | -0.01 |
| TSHI | 0.89 | 0.14 | 0.30 | 0.91 | 0.97 | 1.00 | -0.16 |
| FT3/FT4ratio | 0.14 | 0.55 | -0.68 | -0.40 | -0.01 | -0.16 | 1.00 |

Note: Data are presented as Spearman's rank correlation coefficients (r). TFQI, thyroid feedback quantile-based index; TT4RI, thyrotrophic thyroxine resistance index; TSHI, TSH index.

Table S3. Sensitivity analysis of the association between serum TSH levels and CKD risk using clinical reference ranges.

| TSH Categories | Participants (n) | CKD (n, %) | OR (95% CI) | P-value |
| --- | --- | --- | --- | --- |
| Normal (3.5–5.0 mIU/L) | 385 | 81 (21) | 1.00 (Reference) | - |
| Low (<3.5 mIU/L) | 375 | 73 (19.5) | 0.90 (0.63–1.28) | 0.541 |
| High (>5.0 mIU/L) | 240 | 56 (23.3) | 1.14 (0.77–1.68) | 0.519 |

Note: TSH, thyroid-stimulating hormone; CKD, chronic kidney disease; OR, odds ratio; CI, confidence interval. Data are presented as N (%) unless otherwise specified. Multivariable logistic regression was used to calculate the adjusted ORs and 95% CIs.Models were adjusted for age, sex, smoking status, hypertension, CHD history, diabetes duration, systolic blood pressure, and LDL-C."Normal (3.5–5.0 mIU/L)" group served as the reference for comparison.

Table S4. Association between TSH categories and CKD risk in the PSM-matched cohort

| TSH Category | Total (N) | CKD Cases (N, %) | Adjusted OR (95% CI) | P-value |
| --- | --- | --- | --- | --- |
| Normal TSH | 237 | 36 (15.2%) | 1.00 (Reference) | - |
| Low TSH | 1009 | 117 (11.6%) | 0.69 (0.45–1.06) | 0.090 |
| High TSH | 122 | 33 (27.0%) | 1.67 (0.95–2.95) | 0.076 |

Note: TSH, thyroid-stimulating hormone; CKD, chronic kidney disease; PSM, propensity score matching; OR, odds ratio; CI, confidence interval. Data are presented as N (%) unless otherwise specified. Multivariable logistic regression was used to calculate the adjusted ORs and 95% CIs. Models were adjusted for age, sex, smoking status, hypertension history, CHD history, diabetes duration, systolic blood pressure, and LDL-C. Reference: "Normal TSH" group served as the reference for comparison.

Figure S1. Standardized mean differences of baseline covariates before and after propensity score matching.

The dot plot illustrates the baseline covariate balance between the high TSH group (Q4) and the reference group (Q2) before and after 1:1 propensity score matching. Red hollow dots represent the standardized mean difference (SMD) for each covariate in the original unmatched cohort, and the blue solid dots represent SMD values in the matched cohort. Vertical dashed line at SMD = 0.1 and the solid line at SMD = 0.0 indicate the threshold for acceptable and perfect balance, respectively. All matched covariates achieved good balance, with absolute SMD values consistently below 0.1.

Figure S2. Propensity score distribution of the study cohort.

The density plot illustrates the distribution of propensity scores for the Q4 (high TSH) and Q2 (reference) groups. The significant overlap between the blue (Q2) and red (Q4) curves demonstrates a high degree of common support, indicating that the matching process successfully identified comparable participants across both groups.
